# Management-Associated Risk Factors for Heifer Mastitis before and after Parturition in German Dairy Herds

**DOI:** 10.3390/vetsci10020085

**Published:** 2023-01-23

**Authors:** Philipp Rueben, Martin tho Seeth, Anne Tellen, Volker Krömker

**Affiliations:** 1 Department of Microbiology, Faculty of Mechanical and Bioprocess Engineering, University of Applied Sciences and Arts, 30453 Hannover, Germany; 2 Department of Veterinary and Animal Sciences, University of Copenhagen, 1870 Frederiksberg, Denmark

**Keywords:** mastitis, heifer, risk factor

## Abstract

**Simple Summary:**

The prevention of mastitis (either subclinical or clinical) in heifers has global significance in mastitis control programs. The aim of this study was to identify risk factors associated with a high heifer mastitis rate and to find out whether the period before or after calving provides more risk factors. A pre-partum risk factor was the proportion of udder-healthy cows in the herd. Post-partum risk factors were the type of teat cleaning procedure before milking, teat disinfection, treatment of mastitis in heifers, a body condition score of > 3.0 in fresh heifers, and the combination of a teat cleaning procedure with a teat disinfectant. This outlines the importance of the post-partum period for udder health in heifers. Thus, farmers should focus on post-partum management to improve udder health in heifers.

**Abstract:**

Subclinical mastitis in heifers during early lactation affects udder health, future milk production and, therefore, the risk of premature culling. The aim of this cross-sectional study was to identify pre- and post-partum risk factors associated with a high heifer mastitis rate (HMR), and to find out which period (either pre- or post-partum) contains more risk factors and consequently should be the focus of mastitis control in heifers. A total of 77 herds were included in this study and the potential animal- and farm-related risk factors were recorded during a one-time farm visit. The HMR was provided by the dairy herd improvement test (DHI) as the annual average of the past 11 DHIs. For this study, data were analyzed in two models using generalized linear models. Each model examined the association between possible risk factors and HMR, one including only prepartum risk factors and the other one only post-partum risk factors. One identified pre-partum risk factor was the proportion of udder-healthy cows in the herd. Post-partum risk factors were the type of teat cleaning procedure before milking, teat disinfection, treatment of mastitis in heifers, a body condition score (BCS) of >3.0 in fresh heifers, and the combination of a teat cleaning procedure with a teat disinfectant. The results show the importance of the period shortly after calving for udder health in heifers, as four of the five significant risk factors were identified in this period and three of them were related to the milking process. However, further research with a higher number of herds is needed to minimize individual herd effects.

## 1. Introduction

The prevention of mastitis in heifers is becoming increasingly important, as heifers not only represent a large part of the total herd but also will replace older cows in their future life. Healthy heifers lay the foundation for a long-lasting healthy career as a dairy cow. Due to mastitis prior to the first calving or in the early stages of the first lactation, mammary gland development, future milk production, and udder health may be impaired and the associated risk of premature culling increases [1,2,3].

A distinction is made between clinical mastitis (CM) and subclinical mastitis (SCM). Heifers suffering from CM show visible signs of inflammation. They can either be locally limited to the infected udder quarter (clots in the milk, swelling of the udder quarter) or may affect the general health condition of the animal, e.g., in the form of a fever. An elevated somatic cell count (SCC) > 100,000 cells/mL with the absence of any clinical signs is defined as SCM and is seen as an important benchmark worldwide for evaluating udder health problems in dairy herds [4]. 

Although first-lactating heifers were never milked before, the prevalence of intramammary infections (IMI) varies between 29% and 75% of quarters prior to calving [5]. Nitz et al. [6] found IMI in 19.8% of udder quarters on day three post-partum. The predominant causative agents for IMI (either pre- or post-partum) are non-aureus staphylococci (NAS), but also *Staphylococcus (S.) aureus*, and environmental mastitis pathogens have been associated with IMI in heifers [7].

The prevalence of SCM varies among studies using SCC data as an indicator for SCM depending on the SCC threshold in the dairy herd improvement test (DHI). De Vliegher et al. [8] indicated a prevalence of SCM in post-partum Belgian heifers of 35% using a threshold of 150,000 cells/mL, whereas Bludau et al. [9] found SCM in 20.6% of the heifers in their study using 100,000 cells/mL as a threshold. In Germany, a cow is defined as healthy, in terms of udder health, when the individual animal’s SCC in the DHI is below 100,000 cells per mL [4]. The proportion of heifers exceeding this value in the first DHI after parturition describes the heifer mastitis rate (HMR) and was already proven as a useful tool in recent studies on the subject [9,10,11]. Farms can easily be compared with each other, which makes it useful for HMR herd evaluations, as performed in this study. The heifer mastitis rate is influenced by the time before and after parturition, since a possible infection can also occur in both periods and the first DHI of a heifer occurs between day 5 and 30 post-partum. 

Heifers showing elevated SCC (>100,000 cells/mL) in the first DHI after parturition produced less milk in their first lactation (−98.3 kg) compared to heifers of the same age cohort with ≤100,000 cells/mL [11]. Due to decreased milk production and the higher risk of developing subsequent mastitis, heifers are at risk of being culled prematurely [12,13]. As a consequence, the economic losses due to subclinical mastitis are high; Huijps et al. [14] estimated the cost at €31 (range: €4.29–€82.86) per heifer raised on the farm.

Economic losses, as well as animal welfare and consumer protection, are placing even higher demands on the dairy sector and their farmers. This makes good udder health management more important, which not only includes control measures but, above all, preventive measures. Heifers should therefore be more in the focus of mastitis control in order to take early corrective actions in the udder health management. 

Although much is still unknown about heifer mastitis, several risk factors were already discovered. These can be grouped into three major categories: teat-related risk factors such as loss of the keratin plug prior to calving [15], animal-related risk factors such as increased age at calving [6,16] or the presence of udder and teat edema [17], and herd-specific risk factors such as the calving season [18]. Management practices also seem to have an impact on udder health in heifers just as they do in cows; Piepers et al. [19] indicated that heifers are more likely to contract intramammary infections if they had contact with lactating cows prior to calving, or if no effective fly control was practiced. Nitz et al. [6] described the detaching of milking cups because of kicking off as a risk factor for new infections between days three and 17 post-partum. According to Gösling et al. [20], a prolonged pasture season (>8 months) for pre-partum heifers and pasturing in early spring or late autumn were correlated with a higher HMR.

Also, antibiotic treatment before calving was found to be effective for reducing the risk of IMI after calving [21]. However, this conflicts with today’s guidelines on mastitis control and should not be established as a preventive measure. 

Some studies already identified the period around calving as an important source for risk factors for heifer mastitis [6,15,19,22]. However, to eliminate potential risk factors affecting the heifers’ udder health, it is essential to identify when these risk factors might have an impact on the heifer. Therefore, a distinction should be made between risk factors affecting heifers pre- and post-partum.

The aim of this cross-sectional study was to identify pre- and post-partum risk factors associated with a high HMR at farm level and to derive specific recommendations for better udder health management. 

## 2. Materials and Methods

When studying heifer mastitis, the period around calving reveals many risk factors for heifer mastitis. We accessed risk factors for heifer mastitis which have already been identified in previous studies, but also those that are known for mastitis in multiparous cows, such as management practices. Potential risk factors were recorded both before and after calving to determine whether either the pre- or post-partum time poses more risks for developing mastitis after calving and, therefore, has a greater impact on udder health. 

### 2.1. Farms

**Figure 1:**
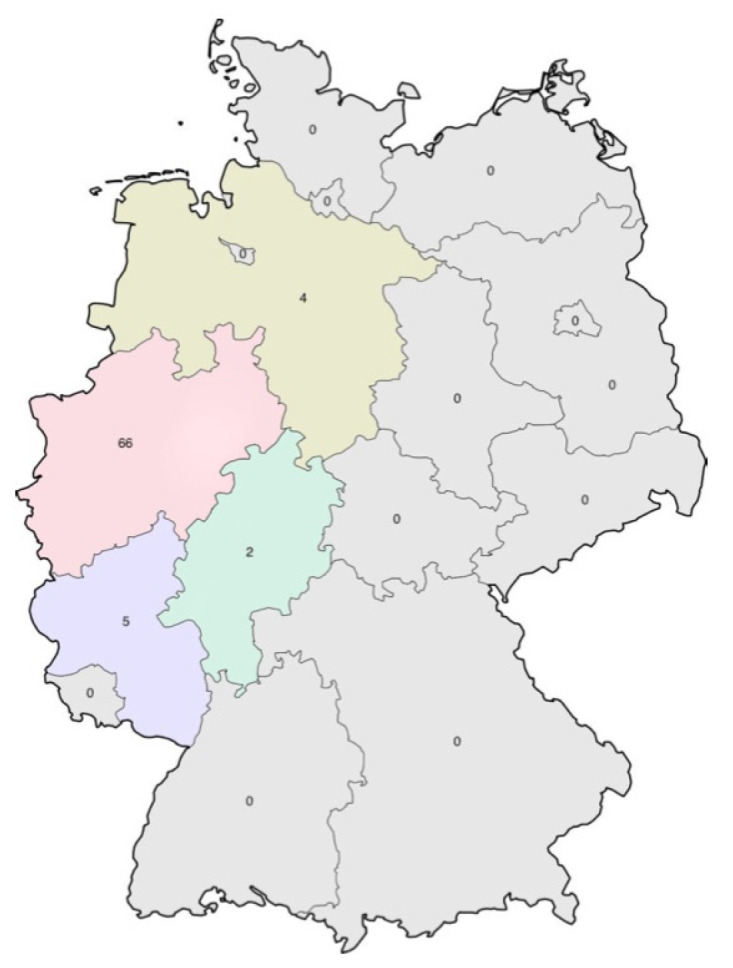
Geographical location in Germany of the surveyed farms. Numbers: number of farms.

Between August 2019 and September 2020, data collection visits took place on 77 farms in the German states of North Rhine-Westphalia, Rhineland-Palatinate, Lower Saxony, and Hesse to assess the risk factors for mastitis in heifers. One visit per farm was conducted. Most of the farms (66) were located in North Rhine-Westphalia. Five farms were located in Rhineland-Palatinate, four in Lower Saxony and two in Hesse (Figure 1). 

The farms chosen for this study were randomly selected to obtain a convenient sample. The inclusion criteria were the availability of a conventional milking system, dairy farms with the predominant breed of black and red Holsteins, and participation in local DHI testing. Herd size varied between 21 and 872 milking cows. Four of the 77 farms were organic farms. All but one farm housed their cows in a cubicle housing system. The cows were milked in a herringbone, rotary, or a side-by-side parlor either two or three times per day. Mean milk yield per herd was 9801 kg (4.07% fat, 3.44% protein)/305 days. Mean herd SCC was 222,000 cells per mL, taken from the DHI of the month of the farm visit.

### 2.2. Animals and Data Collection

During the farm visit, data were collected both in the form of an interview using a questionnaire and by observation. The interview was performed in-person, either during or after the milking process, and included questions about possible management-related risk factors associated with heifer mastitis. The questionnaire consisted of three categories:

1. General questions about farm management, housing, and access to pasture (Table A1);
2. Management-related questions about possible risk factors prior to calving (Table A2);
3. Management-related questions about possible risk factors after calving and risk factors assessed by observation during the milking process (Table 1).

The questions were based on risk factors for heifer mastitis described in the literature, or on risk factors for udder health problems in general. 

The aim of this questionnaire was to collect both quantitative and qualitative data. To obtain comparable data, only close-ended questions were asked. In the case of multiple-choice questions, the farmers were provided a selection of pre-defined responses to choose from. If the response choice was not included, there was the possibility to give an additional response of one’s own choice. Some questions could only be answered with yes or no and sometimes the farmer had to give numeric answers, e.g., when asked about the number of lactating cows on the farm.

The questions about possible risk factors prior to and after calving were mostly related to the housing facilities and management of its bedding, such as housing of pregnant heifers together with dry cows or the frequency of cleaning of the bedding. An overview of those potential risk factors which were treated is shown in Table A2 and Table 1. 

The second part of the farm visit consisted of identifying the potential animal-related risk factors. Therefore, the pregnant heifers prior to calving and heifers in their first month of lactation were chosen for the study. Heifers prior to calving were chosen based on showing udder development/udder edema. They were either housed in a separate group or together with dry cows or in the calving pen. Their Body Condition Score (BCS) was assessed visually using the 1–5 scale as described by Edmonson et al. [23], ranging from 1 (severe under-conditioning) to 5 (severe over-conditioning) with 0.25 increments. For the statistical analysis, three categories were formed, each describing the percentage of heifers with the corresponding BCS (Category 1: BCS ≤ 3; category 2: BCS ≥ 3.25–3.5; category 3: BCS > 3.5). In addition, the heifers’ udder hygiene was evaluated using the scale as described by Schreiner et al. [24]. Each udder was assigned to a category depending on its degree of contamination: 1 (free of dirt), 2 (slightly dirty: 2–10% of surface area), 3 (moderately covered with dirt: 10–30% of surface area), and 4 (covered with caked on dirt: >30% of surface area).

Since the early stage of lactation was of interest for this study, animal-related risk factors were assessed on heifers up to 30 days in lactation. Studies focusing on bacteriological cultures chose thresholds closer to calving (up to 17 days in milk) [6], but for this study, no bacteriological examination was performed, and the focus was more on management. The higher threshold of 30 days after calving was chosen because heifers participate in their first DHI within this timeframe and are included in the HMR.

The fresh heifers were visually characterized during the farm visit regarding body condition and udder hygiene. Furthermore, their teat end condition was evaluated. A scoring system developed by Neijenhuis et al. [25] and simplified for field evaluations by Mein et al. [26] was used. The teat end condition was assigned to one of four groups: teat ends not showing any hyperkeratosis (NR, no ring) were distinguished from teats showing smooth or slightly rough (SR), rough (R), or very rough (VR) hyperkeratosis. 

The severity of udder edema was assessed using a 5-point scale ranging from 1 (no edema) to 5 (extremely severe) [27], and lameness was recorded using the scoring system by Sprecher et al. [28] ranging from 1 (normal) to 5 (severely lame). 

During the milking process, the milking management practices were observed. To evaluate the quality of the teat cleaning procedure, the teat contamination with dirt or manure was determined using the Teat Cleanliness Scorecard by Cook et al. [29]. After the teat had been cleaned by the milker, and before the milking equipment was attached to the animal, each teat was wiped with a white swab and the amount of dirt or manure remaining on the swab was assessed and then assigned to one of the following four categories: 1. (clean: no manure, dirt or dip), 2. (dip present: no manure or dirt), 3. (small amount of dirt and manure present), and 4. (larger amount of dirt or manure present). For the latter parameter, not only heifers but at least 50% of the milking herd was examined.

Also, the teat cleaning procedure was observed (Table 1). We distinguished between the type of material used for the teat cleaning (paper cloths vs. reusable cloths vs. udder shower vs. teat scrubber). In the final model, these were categorized into dry and moist teat cleaning procedures. Furthermore, the use of pre- and post-teat disinfectants, disinfection of the milking cluster, and stress indicators, such as detaching of milking cups because of kicking off or air infiltration in the milking cups, were recorded. Once again, not only heifers but at least 50% of the milking herd was included in this parameter.

Since we aimed to differentiate between risk factors for either the pre- or post-partum periods, data at heifer and farm level were collected separately for heifers before and after parturition. This resulted in two sets of data, each containing the risk factors either related to the period prior to or after parturition. When a potential risk factor was relevant for both periods/heifers, it was included in both datasets.

### 2.3. Heifer Mastitis Rate

In order to study heifer mastitis and to gain an impression of the udder health situation in heifers on a farm, the HMR was used. This was already proven as a useful tool in recent studies on the subject [9,10,11]. Farms can easily be compared with each other, which makes it useful for herd evaluations such as in the present study. The HMR describes the percentage of heifers with >100,000 somatic cells per mL milk in the first DHI test after parturition. Heifers from day 5 to 30 after parturition were assessed. 

The HMR were recorded in the monthly performed DHI test. For the final model, the average HMR of the past 11 months before visiting the farm was used.

Data for CM were not available. 

### 2.4. Statistical Analysis

The collected data from the interviews and the observations of the animals were collected in Microsoft Excel and analyzed with the program SPSS28.0, SPSS Inc., Chicago, IL, USA with the herd as the statistical unit. The association between the HMR and possible risk factors (independent variables) was examined with generalized linear models after pre-screening for variable selection in univariable analysis. The normal distribution of the outcome variable HMR was tested and confirmed using the Kolmogorov–Smirnov test. The relationship between the HMR and the independent variables was first determined using appropriate univariable parametric test procedures. Independent variables associated with the dependent variable at *p* ≤ 0.1, except for predictors in the same model, which indicated a correlation of r > 0.70 with one another (to avoid multicollinearity, no variables were excluded) were submitted to generalized linear mixed models with an identity link. 

The multivariable analysis was performed using a backward stepwise selection and an elimination procedure until each independent variable had a *p*-value of ≤0.05. Confounding effects were monitored by observing regression coefficient changes. Variables that modified regression coefficients by >20% were considered confounding factors. No confounding was observed. The models were evaluated using the Akaike information criterion (AIC), where an AIC closest to zero was used as final model. In the final model, all biologically credible two-way interactions were tested but eliminated again due to lack of significance. Model fit was evaluated by checking the normality of the residuals. Least square means from the model were calculated. 

## 3. Results

### 3.1. Study Herds

This study aimed to identify potential risk factors for heifer mastitis at herd- and heifer-level. The mean herd size of the 77 farms included in this study was 177 lactating cows on the day of the farm visit. The mean milk yield per herd was 9801 kg (4.07% fat, 3.44% protein)/305 days. The mean herd SCC was 222,000 cells per mL, taken from the DHI of the month of the farm visit. The mean age at first calving was 26.6 months. 

### 3.2. Statistical Results

#### 3.2.1. Pre-Partum Risk Factors

In the model regarding risk factors prior to parturition, 77 farms were included, and the average HMR was 29.691% (Table 2). One significant risk factor could be identified: the proportion of udder-healthy cows in the herd was significantly associated with the HMR (Table 3). With a mean proportion of udder-healthy cows of the studied herds of 59% (Table 2), an increase in udder-healthy cows of 1% was associated with a decrease in HMR by an average of 0.374% (*p* = 0.004) (Table 3).

#### 3.2.2. Post-Partum Risk Factors

In the model regarding risk factors after parturition, 71 farms were included, and the average HMR was 29.481% on these farms. Continuous and categorical variable information is provided in Table 4 and Table 5. Five variables were significantly associated with HMR (Table 6). Estimated marginal means describing the differences between the categorical risk factor (independent variable) associated with the HMR are presented in Table 7. In a further analysis, two significant differences between the subgroups of the risk factors could be identified (Table 8). 

The body condition in fresh heifers was significantly associated with the HMR. Herds with a larger proportion of fresh heifers showing a BCS ≤ 3 showed a lower HMR compared to herds where the proportion of fresh heifers showing a BCS ≤ 3 was smaller. The HMR decreased by 0.13% when the proportion of heifers showing BCS ≤ 3 increased by 1% (*p* = 0.002) (Table 8).

As in the model containing only pre-partum risk factors, the proportion of udder-healthy cows in the herd was a risk factor in the post-partum model, although in a further analysis, it was no longer significant.

Herds performing a moist teat cleaning procedure (by using either an udder shower, a teat scrubber, or moist udder cloths) showed an average HMR of 30.1%. Herds performing a dry teat cleaning procedure (using dry reusable cloths or paper cloths) had an average HMR of 20.3%. In the herds where no teat cleaning procedure was performed, the HMR was 20.2%. This meant that herds performing a moist teat cleaning procedure had a significantly higher HMR compared to herds using a dry procedure (*p* < 0.001) (Table 8).

Farms which treated mastitis in fresh heifers using only intramammary treatment (in the infected udder quarter) showed an average HMR of 29.2%. Farms where heifers were treated only by intramuscular antimicrobial injections showed an average HMR of 20.1%. Herds not using any kind of treatment showed an average HMR of 34.1%. Herds using both practices in combination (systemic and local treatment) had an average HMR of 10.8%. The HMR of herds being treated only locally was 18.37% higher compared to herds using both practices (*p* = 0.052) (Table 8).

The use of a teat disinfectant was significantly associated with the HMR (Table 6). The HMR between the herds varied depending on the active ingredient of the teat dip, although no significant differences could be seen between the subgroups in the final generalized linear model (Table 8). A total of 43.7% of the farms used an iodine teat disinfectant, 19.7% used chlorine dioxide, 18.3% used lactic acid, and 4.2% used chlorhexidine. Compared to herds using lactic acid, herds using no teat dip had a 5.95% lower HMR (*p* = 0.351), herds using chlorine dioxide had a 12.61% lower HMR (*p* = 0.196), herds using chlorhexidine had a 3.13% lower HMR (*p* = 0.749), and herds using an iodine-based teat dip had a 5.61% lower HMR (*p* = 0.240) (Table 8).

Interactions between the type of teat cleaning procedure and the active ingredient of the teat disinfectant were also significantly associated with the HMR (Table 8). It would appear that a moist teat cleaning procedure in combination with a teat disinfectant (e.g., chlorine hexidine) was most effective because the HMR decreased by 42.766% (*p* = 0.013) (Table 8).

## 4. Discussion

This study aimed to identify potential risk factors for heifer mastitis on dairy farms located in North Rhine-Westphalia, Rhineland-Palatinate, Lower Saxony, and Hesse and it also aimed to show whether either the pre- or post-partum period contains more risk factors.

Monitoring SCC in lactating cows is part of mastitis control programs worldwide [4,8,9,10,11]. An elevated SCC above a specific threshold indicates SCM [4]. In this study, DHI data provided the HMR, in which the threshold of 100,000 cells/mL for SCM is used as in other studies on the subject [4,6,9,22]. Higher SCC thresholds for SCM may also be valid, as it is used in other countries [8,9], although a recent study confirmed the validity of the threshold of 100,000 cells/mL SCC [11]. De Vliegher et al. [16] found out that an increase in SCC up until day 5 post-partum is physiological. This potential error was excluded in our study, since only heifers from day 5 post-partum were included in the DHI. 

IMI might also be used as an indicator for mastitis in heifers, but we did not perform a microbiological analysis in the present study. Therefore, clinical infections and the number of infected udder quarters were not assessed. 

For this study, the farms were selected based on their agreement to participate in the study. Only two farmers declined. No preselection was made. Our findings in this study may be valid for other farms in the same region, although transferring the results should be interpreted with caution. The average herd size of 177 cows in milk was larger than the average herd size of herds in North Rhine-Westphalia participating in the DHI in 2019 (96.9 cows) [30] and in Rhineland-Palatinate in 2019 (84.6 cows) [31]. The HMR (29.7%) was slightly below average compared to the average HMR of herds in North Rhine-Westphalia (30.3%) [32]. However, participation in the DHI is voluntary in Germany, which is why the actual average herd size and HMR may well differ. In 2019, 1204 farms with a collective total of 101,815 cows did participate in the DHI in Rhineland-Palatinate, while in the same year, 675 farms with a collective total of 18,654 cows did not participate in the DHI [33]. 

Also, herd-specific risk factors such as season or climate differ among regions and could also have an impact, but they were not considered in this study. To ensure repeatability of interview data and to minimize interview-bias, farm visits and interviews were always conducted by the same person. The interviewee was not necessarily the farmer but could also be the herd manager.

### 4.1. Pre-Partum Risk Factors

Pre-partum management of pregnant heifers can favor IMI leading to SCM in the beginning of their first lactation. As our study shows, the proportion of udder-healthy cows in a herd is associated with the HMR. In Germany, a cow is defined as udder-healthy if SCC is below 100,000 cells/mL, and the percentage of cows not exceeding this threshold is assessed in the DHI. These data can be used to describe the udder health situation on a farm. This is in agreement with earlier findings. Piepers et al. [19] stated that a high average herd milk SCC (>200,000 cells/mL) increases the risk of heifers having intramammary infections with contagious pathogens. Also, heifers exhibited higher SCC when they came from herds with high bulk-milk SCC (BMSCC) [16]. Infected udder quarters of lactating and dry cows are a possible source for the transmission of pathogens to heifers. The transmission could possibly take place during the milking process or when heifers are housed in the same barn as dry cows, which is why some studies concluded that the housing of heifers together with lactating cows should be avoided [19]. In the present study, this consideration was not significantly associated with the HMR. It should be considered that the herd milk SCC also contains the data from the heifers’ SCC, although their proportion should not be too large, since the number of fresh heifers in a herd is low. 

### 4.2. Post-Partum Risk Factors

The pre-milking teat cleaning procedure is established in mastitis control programs to reduce bacterial load on the teat skin and, therefore, prevent pathogens from entering the teat canal and causing IMI. In this study, farms using moist teat cleaning devices (moist cloths or an udder shower) had a significantly higher HMR (+9.8%) than farms using dry devices. In 2006, Magnusson et al. [34] compared different pre-milking teat cleaning methods and their effect on bacterial spores in milk. Up to 50% fewer spores were found in the milk when teats were cleaned using a dry paper towel in comparison with no teat cleaning at all. By using moist towels, the concentration of spores in milk could be reduced by up to 74%. In another study, the effect of different types of teat cleaning methods on the teat microbial load was determined. Gibson et al. [35] found all the cleaning techniques were able to reduce the microbial load on the teat, although the chlorine wash and dry was the most effective. If reusable cloths are used for the teat cleaning procedure, it is recommended to use one cloth per cow (=4 teats) and to wash the cloths afterwards. Under unsanitary circumstances, e.g., using one cloth for more than one cow or an ineffective washing procedure of the cloths, bacterial growth can occur and pathogens can be transmitted to the teat through the cloths. This could be a possible explanation for the worse HMR in herds using moist teat cleaning devices. On the other hand, Hohmann et al. [36] found no increase in bacterial loads when more than one cow was cleaned per wipe. However, efficiency of teat cleaning not only depends on the device used for cleaning, but also on how thorough the person is when cleaning the teat. To exclude this effect, we evaluated the teat cleanliness with the teat cleanliness scorecard. Baumberger et al. [37] assessed the mesophilic bacteria load to describe the efficacy of the pre-cleaning, but the allocation of the score seemed easier to integrate into the milking routine. There was no significant association between the efficiency of the teat cleaning and the HMR in our study. 

Another part of mastitis control is teat disinfection, which was significantly associated with the HMR at the beginning of the statistical analysis (Table 6). Although no significant connection between the HMR and the active ingredient of the teat dip was shown in further analysis, the HMR varied slightly between farms using different teat dips (Table 7). Although one study showed that the reduction in bacterial growth differed among different teat disinfectants [38], the use of some kind of teat disinfection is recommended to ensure high milk quality. 

It was also found that a lower BCS in fresh heifers was associated with a lower HMR. Heifers with increased age at first calving tend to have higher BCS at calving, which lead to metabolic stress due to weight loss, higher beta hydroxybutyrate blood concentration, and, therefore, a higher risk for mastitis in early lactation [39]. Bassel et al. [40] were able to show that with an increased age at calving, heifers had a higher risk for developing IMI with *S. aureus* and environmental pathogens. Nitz et al. [6] described an increased risk of IMI with non-*aureus* staphylococci and coryneforms 17 +/− 3 days after calving in heifers calving at an older age. The association with the age at first calving makes the body condition and its evaluation in heifers and cows an easy and useful tool in udder health management. In our findings, the average age at first calving of all farms with available data was 26.6 months. 

Although prevention of mastitis should be a priority in mastitis control, therapeutic intervention according to the latest standards is not any less important. In order to remove the agent causing mastitis and to prevent infections of other animals, antimicrobial therapy can be indicated. Antimicrobial treatment of subclinical mastitis in lactation is not recommended, while clinical cases should be treated depending on their severity. Mild and moderate cases of CM should be treated locally by administering an antibiotic injection into the infected udder quarter, while severe cases with systemic symptoms (such as fever or septicemia) should be treated systemically [41]. In the present study, the farmers were asked how they treated mastitis (either clinically or sub-clinically) in heifers. This variable was significantly associated with the HMR (*p* = 0.038), although no significant association was found between the subgroups (local, systemic treatment, or both). The HMR of herds being treated only locally (n = 61) was 18.37% higher compared to herds (n = 1) where both practices were used (*p* = 0.052). This might be an effect of the better distribution of the injected drug. The substance used for antimicrobial treatment was penethamate hydroiodide, which is able to penetrate through the blood-milk barrier and therefore achieve high concentrations in all udder quarters. Due to the limited number of herds (n = 1), the risk factor could not be evaluated properly. This study only focused on mastitis treatment after parturition. The farmers were also asked if they used antibiotic treatment in heifers prior to calving, but all of them answered negatively. Although several studies showed that the antimicrobial treatment in young heifers can be beneficial, with regard to today’s guidelines on mastitis control, it should not be established as a standard preventive measure.

Based on the data provided by the DHI, we were unable to identify the time of infection (either pre- or post-partum) leading to increased SCC on the first testing day. However, our results suggest the importance of the time after parturition for udder health in first lactating heifers, since most of the risk factors significantly associated with the HMR were assessed during the post-partum period. This is in agreement with Nitz et al. [6]. Other studies found the following risk factors for the time prior to calving: loose-housing systems during pregnancy, juvenile inter-sucking, organic bedding material prior to calving [22], open teat canal prior to calving [15], BMSCC, milking system, culling rate due to udder health disorders, and having a standard operating procedure for colostrum feeding of calves [42]. Management practices, either related to the pre- or post-partum period, can be key factors in mastitis control programs on dairy farms. Nevertheless, to improve the udder health in heifers on the farms considered in this study, the farmers should focus on post-partum management. 

### 4.3. COVID-19

Farms were surveyed before and during the COVID-19 pandemic. COVID-19 was officially declared as a global pandemic 11 March 2020 [43] and farms were visited between August 2019 and September 2020. However, the pandemic did not affect data collection because we were able to contact farmers and visit farms without hindrance. Later in the pandemic, the medical supply chain was disrupted and the way it was managed changed [44]. However, during our study, there was no shortage of medical supplies, such as the relevant disinfectants, gloves, or even bedding material. The supply shortage may have affected the farms later on in the pandemic.

## 5. Conclusions

This study outlined herd-level management practices associated with the HMR. In the statistical analysis of the pre-partum risk factors, the proportion of udder-healthy cows in a herd were significantly associated with the HMR. In the post-partum model, the type of teat cleaning procedure, teat disinfection, mastitis treatment, a BCS ≤ 3.0 in fresh heifers, and the combination of a teat cleaning procedure and a teat disinfectant were significantly related to the HMR. However, in further analysis where subgroups were compared to each other, the differences between the subgroups were no longer significant, except for the moist teat cleaning procedure (*p* ≤ 0.001) and interactions between the teat cleaning procedure and the active ingredient of the teat dip (Table 8). The results show that the time after parturition influences the udder health of heifers. Thus, focus should be placed on that specific period when investigating risk factors for heifer mastitis. However, as only 77 farms were examined in this study, more herds should be included in future investigations, so that the findings will be better substantiated scientifically.

## Figures and Tables

**Table 1:** Management-related questions about possible risk factors after calving and risk factors assessed by observation during the milking process.

Independent Variable	Recording Method	Description/Classification	Break down Categories Final Model
Teat cleaning	Observation	Whether or not teats get cleaned before the milking unit is attached	Yes vs. no
Teat cleaning device	Observation	The kind of implement used for teat cleaning	Paper vs. reusable cloth vs. udder shower vs. teat scrubber
Type of teat cleaning	Observation	Whether the cloth is dry or moist	Dry vs. moist
Number of cows the cloth is used on	Observation	Number of cows one cloth is used on	
Disposable gloves	Observation	Whether or not the milker uses disposable gloves	Yes vs. no
Disinfecting the hands/gloves	Observation	Whether or not the hands/gloves are disinfected during and between the milking process	Yes vs. no
Air infiltration	Observation	Proportion of air infiltrations in percent	
Detaching of milking cups because of kicking off	Observation	Proportion of detached cups in percent	
Defecation	Observation	Proportion of defecating cows in percent	
Intermediate disinfection	Observation	Whether or not the milking cups are disinfected between cows	Yes vs. no
Active ingredient	Interview	Active ingredient of the intermediate disinfection	
Type of intermediate disinfection	Observation	Whether the milking cups are dunked in disinfection or the disinfection is sprayed on	Dunked vs. sprayed on
Teat dip	Observation	Whether or not a teat dip is used	Yes vs. no
Active ingredient	Interview	Active ingredient of teat dip	
Type of teat dip	Observation	Time of teat dipping	Pre vs. post vs. both
Staff in milking parlor	Interview	Number of persons present in milking parlor	
Mastitis therapy in heifers	Interview	Whether clinical heifer mastitis is treated with antibiotics, homeopathic or with both	Antibiotics vs. homeopathic vs. both
Mastitis therapy in heifers	Interview	Whether heifer mastitis is treated systemically, locally or both	Systemic vs. local vs. both
Housing of the heifers in milk	Interview		Together with cows vs. in their own group
Moving fresh heifers to milking herd/group	Interview	Time period the fresh heifers stay in a separated group after calving before being moved to the milking herd expressed in days	
Udder edema	Interview	Whether or not udder edema is treated in any way	Yes vs. no
Type of cubicle for fresh heifers	Interview	What kind of cubicle is provided for the fresh heifers?	Deep-bedding cubicles vs. cubicles with rubber mats vs. deep litter barn vs. slatted flooring
Raking the cubicles clean where the fresh heifers are housed	Interview	How often are the cubicles raked clean?	Daily vs. weekly vs. if required vs. never
Bedding material	Interview	What kind of bedding material is used?	Manure solids vs. horse manure vs. straw vs. sawdust vs. none
Adding new bedding material	Interview	Interval of adding new bedding material expressed in days	≤3 vs. >3 ≤6 vs. >6 ≤9 vs. >9
Cover layer on top of bedding material	Interview	Whether or not a layer is added on top of the bedding material	Yes vs. no
Shortage of bedding material	Interview	Whether or not there was a shortage of bedding material	Yes vs. no
Cleaning of drinking trough	Interview	Interval of cleaning expressed in days	Zero vs. 1 vs. 2 vs. ≥3

**Table 2:** Continuous variable information.

		N	Minimum	Maximum	Mean	Std. Deviation
Dependent variable	HMR ^1^	77	7.1	100.0	29.691	13.187
Covariate	Proportion of udder healthy cows in herd	77	32.4	80.6	59.029	10.973

^1^ Heifer mastitis rate.

**Table 3:** Generalized linear model describing pre-partum risk factors (independent variable) associated with HMR1 (dependent variable).

Independent Variable	B ^2^	SE ^3^	95% CI ^4^		*p*-Value
			Lower	Upper	
Proportion of udder-healthy cows in herd	−0.374	0.1301	−0.630	−0.119	0.004

^1^ Heifer mastitis rate. ^2^ Regression coefficient. ^3^ Standard error. ^4^ Confidence interval.

**Table 4:** Continuous variable information.

		N	Minimum	Maximum	Mean	Std. Deviation
Dependent variable	HMR ^1^	71	7.1	100.0	29.365	13.440
Covariate	Percentage of fresh heifers showing BCS ^2^ ≤ 3 of all fresh heifers	71	0.0	100.0	30.707	29.481
	Percentage of udder-healthy cows	71	37.4	80.6	59.575	10.754

^1^ Heifer mastitis rate ^2^ Body Condition Score.

**Table 5:** Categorical variable information.

Independent Variable	Categories	N	%
Type of teat cleaning procedure	None	20	28.2
	Moist	30	42.3
	Dry	21	29.6
	Total	71	100.0
Active ingredient of teat dip	None	10	14.1
	Chlordioxide	14	19.7
	Chlorhexidine	3	4.2
	Iodine	31	43.7
	Lactic acid	13	18.3
	Total	71	100.0
Treatment of mastitis in heifers	None	3	4.2
	Intramuscular (systemically)	6	8.5
	Locally	61	85.9
	Both (locally and systemically)	1	1.4
	Total	71	100.0

**Table 6:** Independent variables significantly associated with HMR ^1^—tests of model effects.

Independent Variable	Wald Chi-Square	Df ^2^	*p*-Value
Type of teat cleaning procedure	8.836	2	0.012
Active ingredient of teat dip	23.355	4	0.000
Treatment of mastitis in heifers	8.436	3	0.038
BCS ^3^ ≤ 3 in fresh heifers	9.726	1	0.002
Interaction between teat cleaning procedure and teat disinfection	22.799	8	0.004

^1^ Heifer mastitis rate. ^2^ Degrees of freedom. ^3^ Body Condition Score.

**Table 7:** Estimated marginal means describing the differences between categorical risk factor (independent variable) associated with HMR ^1^ (dependent variable).

Independent Variable and Categories	Mean	SE ^2^	95% CI ^3^	
			Lower	Upper
Type of teat cleaning procedure				
None	20.240	3.6156	13.154	27.326
Moist	30.078	3.4688	23.280	36.877
Dry	20.307	4.4641	11.558	29.057
Active ingredient of teat dip				
None	20.485	4.6150	11.440	29.530
Chlordioxide	19.523	5.1262	9.476	29.570
Chlorhexidine	18.678	7.1430	4.678	32.678
Iodine	23.044	3.5411	16.103	29.984
Lactic acid	35.979	4.2922	27.566	44.391
Treatment of mastitis in heifers				
None	34.081	8.6701	17.088	51.074
Intramuscular (systemically)	20.066	4.5018	11.242	28.889
Locally	29.198	2.0458	25.188	33.207
Both (locally and systemically)	10.823	9.5970	−7.986	29.633

^1^ Heifer mastitis rate. ^2^ Standard error. ^3^ 95% confidence interval.

**Table 8:** Final generalized linear model describing post-partum risk factors (independent variables) associated with HMR ^1^ (dependent variable).

Independent Variable	Categories	B ^2^	SE ^3^	95% CI ^4^		*p*-Value
				Lower	Upper	
Teat cleaning procedure performed	None	−4.145	5.994	−15.893	7.603	0.489
	Moist	34.783	6.3784	22.282	47.285	<0.001
	Dry	0^a^				
Active ingredient of teat dip	None	−5.954	6.383	−18.464	6.557	0.351
	Chlorine dioxide	−12.606	9.739	−31.695	6.483	0.196
	Chlorhexidine	−3.126	9.785	−22.304	16.052	0.749
	Iodine	−5.610	4.779	−14.976	3.757	0.240
	Lactic acid	0 ^a^				
Mastitis therapy in fresh heifers	None	23.257	13.584	−3.367	49.881	0.087
	Systemically	9.242	10.427	−11.194	29.679	0.375
	Locally	18.374	9.449	−0.144	36.893	0.052
	Both	0 ^a^				
BCS ^5^ ≤ 3.0 in post-partum heifers		−0.130	0.042	−0.212	−0.048	0.002
No teat cleaning procedure × no dipping		3.904	9.086	−13.904	21.712	0.667
No teat cleaning procedure × chlorine dioxide teat dip		10.979	11.956	−12.455	34.412	0.359
No teat cleaning procedure × chlorhexidine teat dip		0.241	16.810	−32.705	33.188	0.989
No teat cleaning procedure × iodine teat dip		5.267	7.424	−9.283	19.817	0.478
No teat cleaning procedure × lactic acid teat dip		0^a^				
Moist teat cleaning procedure × no dipping		−32.524	9.9566	−52.038	−13.009	0.001
Moist teat cleaning procedure × chlorine dioxide teat dipping		−22.526	11.4143	−44.898	−0.154	0.048
Moist teat cleaning procedure × chlorhexidine		−42.766	17.2537	−76.583	−8.950	0.013
Moist teat cleaning procedure × iodine teat dip		−27.243	7.5441	−42.029	−12.457	<0.001
Moist teat cleaning procedure × lactic acid		0 ^a^	.	.	.	.
Dry teat cleaning procedure × no dip		0 ^a^	.	.	.	.
Dry teat cleaning procedure × chlorine dioxide		0 ^a^	.	.	.	.
Dry teat cleaning procedure × chlorhexidine		0 ^a^	.	.	.	.
Dry teat cleaning procedure × iodine		0 ^a^	.	.	.	.
Dry teat cleaning procedure × lactic acid		0 ^a^	.	.	.	.

^1^ Heifer mastitis rate. ^2^ Regression coefficient. ^3^ Standard error. ^4^ 95% confidence interval. 5 Body Condition Score. ^a^ set to zero because this parameter is redundant.

## Data Availability

Not applicable.

## Appendix A

**Table A1:** General questions raised in the interview concerning farm management, housing, and access to pasture.

Independent Variable	Description/Classification	Break down Categories Final Model
Number of lactating cows	Number of lactating cows on the day of the farm visit	
Type of production	Whether the farms practice conventional or organic farming	Conventional vs. organic
Times cows are milked per day	Two–three times	Two vs. three
Water source	Drinking water source in the stall	Well water vs. drinking water vs. sea level
Type of milking parlor		
Calving season	Whether or not cows only freshen in specific seasons	Yes vs. no
Access to pasture	Whether or not the heifers and/or cows have access to pasture	Yes vs. no
Access to pasture	When do the cows/heifers gain access to pasture?	During the day vs. during the night vs. day and night
Time access to pasture per day	Expressed in hours per day	
Duration of the pasture season	Expressed in months per year	
Age at first access to pasture	Age at first access to pasture expressed in months	
Start of pasture season	Expressed in name of month the pasture season starts	
End of pasture season	Expressed in name of month the pasture season ends	
Water source	Water source on pasture	Well water vs. drinking water vs. sea level
Consolidation of livestock trails	Whether or not livestock trials are consolidated	Yes vs. no
Consolidation material of livestock trials		Attached vs. loose vs. perforated
Fly control strategy	Whether or not a fly control strategy is practiced on the farm	Yes vs. no
Fly control strategy	Type of fly control strategy	Pour on vs. ear tags vs. both

**Table A2:** Management-related questions raised in the interview about possible risk factors prior to calving.

Independent Variable	Description/Classification	Break down Categories Final Model
Housing of pregnant heifers	Location of pregnant heifers prior to calving that were selected for the study	Together with dry cows vs. own group vs. on pasture vs. on a rearing farm
Type of cubicle for pregnant heifers	What kind of cubicle is provided for the pregnant heifers?	Deep-bedding cubicles vs. cubicles with rubber mats vs. deep litter barn vs. slatted flooring
Raking the cubicles clean where pregnant heifers are housed	How often are the cubicles raked clean?	Daily vs. weekly vs. if required vs. never
Bedding material	What kind of bedding material is used?	Manure solids vs. horse manure vs. straw vs. sawdust vs. none
Adding new bedding material	Interval between adding new bedding material expressed in days	≤3 vs. >3 ≤6 vs. >6 ≤9 vs. >9
Cover layer on top of bedding material	Whether or not a layer is added on top of bedding material	Yes vs. no
Shortage of bedding material	Whether or not there was a shortage of bedding material	Yes vs. no
Cleaning of drinking trough	Cleaning interval expressed in days	Zero vs. 1 vs. 2 vs. ≥3
Calving area	Whether or not heifers and cows share a calving area	Together vs. separated
Moving heifers to calving area	Timepoint when heifers were moved to calving area before calving expressed in days	
Mucking out the calving area	Mucking out interval of calving area expressed in days	
Disinfection of calving area	Whether or not calving area is disinfected after cleaning	Yes vs. no
Juvenile inter-sucking among heifers	Number of heifers inter-sucking currently	
Pre-fresh diet	Whether or not heifers receive a pre-fresh diet prior to calving	Yes vs. no
Pre-fresh diet	Time period the pre-fresh diet is available for heifers expressed in days	
Mineral supplements	Type of mineral supplementation	
Vitamin E/selenium	Whether or not vitamin E/selenium is injected	Yes vs. no
Udder clipping	Whether or not udders are clipped	Yes vs. no
Use of teat sealant and/or antibiotic treatment prior to calving	Whether or not teat sealant and/or antibiotic treatment is used in heifers prior to calving	Yes vs. no
